# Estrogenic control of germ cell differentiation in medaka: independence of early sex dimorphism from zygotic estrogen and receptor signaling

**DOI:** 10.3389/fendo.2025.1769798

**Published:** 2026-01-19

**Authors:** Yuta Sakai-Yamada, Taijun Myosho, Daichi Kayo, Shinji Kanda, Tohru Kobayashi

**Affiliations:** 1 Laboratory of Molecular Reproductive Biology, Institute for Environmental Sciences, University of Shizuoka, Shizuoka, Japan; 2 Graduate School of Pharmaceutical and Nutritional Sciences, University of Shizuoka, Shizuoka, Japan; 3 Division of Applied Biosciences, Graduate School of Agriculture, Kyoto University, Kyoto, Japan; 4 Department of Biology, Faculty of Agriculture and Life Science, Hirosaki University, Hirosaki, Japan; 5 Laboratory of Physiology, Atmosphere and Ocean Research Institute, The University of Tokyo, Kashiwa, Chiba, Japan

**Keywords:** estrogen receptor, germ cell number, medaka, null, oogenesis, sex differentiation

## Abstract

**Background:**

Estrogen signaling is essential for ovarian differentiation in vertebrates, but its developmental onset and specific roles during early gonadogenesis remain unclear. In medaka (*Oryzias latipes*), the first morphological sex difference appears as a higher germ cell number in XX compared with XY embryos before hatching. Recently, we demonstrated that zygotically synthesized estrogen was dispensable for early germ cell sex difference but essential for subsequent oocyte meiotic progression and ovarian fate maintenance. Nevertheless, whether these phenomena depend on maternal estrogen or zygotic estrogen signaling mediated by nuclear estrogen receptors (nEsrs) is unknown.

**Objective:**

To clarify the receptor-level requirement of estrogen signaling, we generated *esr1/2a/2b* triple knockout (ΔnEsrs) medaka using CRISPR/Cas9 genome editing. By comparing these receptor-deficient mutants with our previous ligand-deficient (Δ*cyp19a1a/1b* double knockout) model, we aimed to determine whether estrogen signaling is involved in the establishment of the early germ cell number difference or acts later to control meiotic progression and ovarian maintenance.

**Methods:**

We established ΔnEsrs medaka using CRISPR/Cas9 and analyzed gonadal histology, germ cell kinetics, and expression of steroidogenic enzyme genes, sex differentiation-related genes, and oocyte-specific expressed genes during early development.

**Results:**

ΔnEsrs mutants displayed normal early germ cell number and sex-specific differences at hatching (0 dph). At 10 dph, diplotene oocytes were markedly reduced, accompanied by significant downregulation of oocyte-specific genes, *figa*, *42sp50*, as well as *cyp19a1a* and *foxl2* mRNA. However, ΔnEsrs did not cause feedback regulation on other hypothalamus-pituitary-gonad (HPG) axis gene expression.

**Conclusion:**

Our results demonstrate that estrogen signaling, both at the ligand and receptor levels, is dispensable for establishing the early germ cell sex difference but essential for subsequent oocyte meiotic progression and ovarian fate maintenance. This establishes a two-step estrogenic control model, redefining the developmental timing of estrogen action during the early phase of gonadal differentiation in vertebrate reproduction.

## Highlights

- nEsr signaling is not required for early germ cell sex dimorphism in medaka.
- nEsr signaling promotes pachytene–diplotene transition in oogenesis.
- Defines a two-step estrogenic control model for vertebrate gonadal differentiation.

## Introduction

Estrogens play crucial roles in vertebrate reproduction, acting through nuclear estrogen receptors (nEsrs) to regulate gonadal differentiation, gametogenesis, and secondary sexual characteristics (1–3). In teleost fish, as in other vertebrates, estrogen signaling is indispensable for ovarian differentiation and maintenance, whereas androgen signaling promotes testicular development (4, 5). Despite these well-established roles, the developmental onset and specific contributions of estrogen signaling to the earliest stages of gonadal sex differentiation remain largely unclear.

In the medaka (*Oryzias latipes*), sex is genetically determined by the Y-linked gene *dmy*, which activates the male pathway through *gsdf* expression in somatic cells (6, 7). One of the earliest morphological sex differences in medaka is sex difference in germ cell number, in which XX embryos exhibit a higher number of germ cells than XY embryos (8, 9). This germ cell number difference represents the first visible dimorphism between the sexes and has long been regarded as a fundamental event in gonadal sex differentiation. However, whether this early difference depends on estrogen signaling, including maternal (egg-derived) and zygotically synthesized estrogen, remains unresolved.

Estrogen production in teleosts is mediated by two aromatase enzymes, *cyp19a1a* (ovarian type) and *cyp19a1b* (brain type), which together establish zygotic estrogen synthesis during early embryogenesis (10–12). Recently, we demonstrated that zygotically synthesized estrogen was dispensable for establishing the early germ cell sex difference but essential for subsequent oocyte meiotic progression and ovarian fate maintenance (13). Specifically, *cyp19a1a/b* double knockout (Δcyp19a1s) medaka exhibited normal germ cell number and sex-specific dimorphism at hatching, but both total germ cell number and diplotene-stage oocytes were markedly reduced at 10 days post hatching (dph). This study revealed that zygotically synthesized estrogen signaling is not required for the formation of the first morphological sex difference but becomes indispensable later for oocyte differentiation and survival. These findings raised a critical question: Does the loss of nEsr signaling recapitulate the same phenotype as the loss of zygotic estrogen synthesis, or does it cause additional effects in gonadal differentiation? Resolving these questions also shed light on the roles of maternal estrogen and estrogen signaling pathway other than nEsr during the early stages of gonadal differentiation in teleosts. In mammals, multiple estrogen receptor isoforms (ESR1 and ESR2) cooperate to mediate estrogen-dependent transcription in ovarian development, and their combined loss leads to severe folliculogenesis defects (14, 15). In teleosts, gene duplication events have produced three nEsr subtypes—*esr1*, *esr2a*, and *esr2b*—that may function redundantly or possess subtype-specific roles (16–18). In medaka, a previous study reported that *esr1*, *esr2a*, and *esr2b* mRNA were detected by RT-qPCR in both sexes on 6 days post fertilization (19). Although this suggests that endogenous estrogen, including maternal and/or zygotically synthesized estrogen, may function in gonadal differentiation and development, the developmental timing and necessity of these receptors for controlling germ cell dynamics remain largely unexplored.

To clarify the receptor-level requirement of estrogen signaling, we generated *esr1/2a/2b* triple knockout (ΔnEsrs) medaka using CRISPR/Cas9 genome editing. By comparing these receptor-deficient mutants with our previous ligand-deficient (Δcyp19a1s) model (13), we aimed to determine whether estrogen signaling contributes to establishing early germ cell number dimorphism, or instead functions later to control meiotic progression and ovarian maintenance.

Here, we show that ΔnEsrs mutants exhibit normal early germ cell numbers and sex-specific differences at hatching, but significantly reduced diplotene oocytes during post-hatching development. These results demonstrate that nEsr-mediated signaling is dispensable for establishing early germ cell sex dimorphism, yet is essential for subsequent oocyte meiosis.

Together with our previous findings from Δcyp19a1s mutants (13), these data establish a two-step zygotic estrogenic control model for medaka gonadal differentiation:

1. an estrogen-independent phase, in which germ cell number and early sexual dimorphism are established by the *dmy–gsdf* axis before hatching; and
2. an estrogen-dependent phase, in which estrogen and nEsr signaling promote the pachytene–diplotene transition and maintain ovarian identity.

This dual-phase framework redefines the developmental onset of estrogen action in vertebrate gonadogenesis and offers new mechanistic insight into how environmental endocrine modulators disrupt reproduction.

## Materials and methods

### Fish

Single knockout (SKO) medaka (d-rR strain, *O. latipes*) of nEsr subtypes (*esr1*, *esr2a*, and *esr2b*), previously reported by Kayo et al. (17), were maintained in aquaria at the University of Shizuoka, Japan, under a controlled 14 h light:10 h dark photoperiod at 27 ± 1 °C. SKO lines were crossbred to establish DKO and TKO medaka, and the resulting knockouts were maintained independently in our aquaria at the University of Shizuoka (SI, **Supplementary Figure S1**: Generation of double and triple nEsr mutants). All animal care and experimental procedures adhered to the Guide for the Care and Use of Laboratory Animals and were approved by the Institutional Committee of Laboratory Animal Experimentation (approval numbers: 688-2303, 689-2303). Medaka fry were euthanized with an overdose of 2-phenoxyethanol (0.5%: Fujifilm Wako, Tokyo, Japan), followed by decapitation, in accordance with the American Veterinary Medical Association (AVMA) Guidelines for the Euthanasia of Animals (2020).

### Sexing

Total genomic DNA was extracted from caudal fin clips or tail muscle tissue using a standard proteinase K digestion method, followed by phenol/chloroform extraction. The genetic sex of each fish was determined via polymerase chain reaction (PCR) targeting the sex-determining gene *dmy*, as previously reported (9, 20).

### Histological assessment of the gonads

Gonadal samples were collected at stage 39 (hatch day: 0 dph, 9 days post-fertilization [dpf]) and at 10 dph, according to the stages of normal development in medaka (21). The fish were anesthetized using 0.5% 2-phenoxyethanol before dissection. The gonads were fixed in Bouin’s fixative solution for 2 h, dehydrated in a graded ethanol series, and embedded in Paraplast Plus (McCormick Scientific, St. Louis, MO, USA). Serial cross-sections (5 μm thick) of the gonads were prepared and stained with Carazzi’s hematoxylin to assess gonadal differentiation. Germ cells in the cross-sections were counted at each differentiation stage as previously described (9, 13). Additionally, the germ cell showing nuclear condensation was classified as an apoptotic cell, whereas this was not verified using other apoptosis markers, such as the TUNEL method and caspase-3.

### RNA extraction and reverse transcription-quantitative PCR

Fish fry were anesthetized with 0.5% 2-phenoxyethanol in dechlorinated water before being preserved in RNAlater reagent (Ambion, Austin, TX, USA) at 4 °C until RNA extraction. Total RNA was extracted from the trunk tissues using the NucleoSpin RNA kit (TaKaRa, Shiga, Japan) following the manufacturer’s instructions. RNA samples were then analyzed using RT-qPCR following previously described procedures (13, 20). The expression levels of target genes are as follows: testis differentiation-related gene (*gsdf*), ovary differentiation-related gene (*foxl2*, and *amhr2*), oocyte-specific expressed genes (*figa*, *scp3*, *bmp15*, and *42sp50*), estrogen synthesizing enzyme genes (*cyp19a1a*, and *cyp19a1b*), steroid synthesizing enzyme genes (*cyp11a*, *cyp17a1*, *and cyp17a2*), androgen-synthesizing enzyme gene (*cyp11b*), HPG axis genes (*gnrh1*, *gnrhr1*, *lhb*, *fshb*, *lhr*, and *fshr*), HPT axis genes (*trh* and *tshba*), and HPS axis genes (*ghrh*, *ghra*, *ghrb*, and *gh1*), were normalized to those of *elongation factor 1 alpha* (*ef1α*). Data are expressed as the mean per individual, correcting for *ef1α* levels. The primer sequences for RT-qPCR were listed in **Supplementary Table S1**. The specificity of each RT-qPCR assay was shown in **Supplementary Figure S2**.

### Statistical analysis

Statistical significance in germ cell number between WT and ΔnEsrs was analyzed by the Student’s t-test, and one-way ANOVA followed by the Tukey-Kramer multiple comparison tests. For RT-qPCR, statistical analyses were performed by one-way ANOVA followed by the Tukey-Kramer multiple comparison tests. All statistical analyses were conducted using Excel (version 4.07; SSRI Inc., Tokyo, Japan).

## Results

### Effects of triple nEsr knockout on germ cell number during sex differentiation

**Figure 1:**
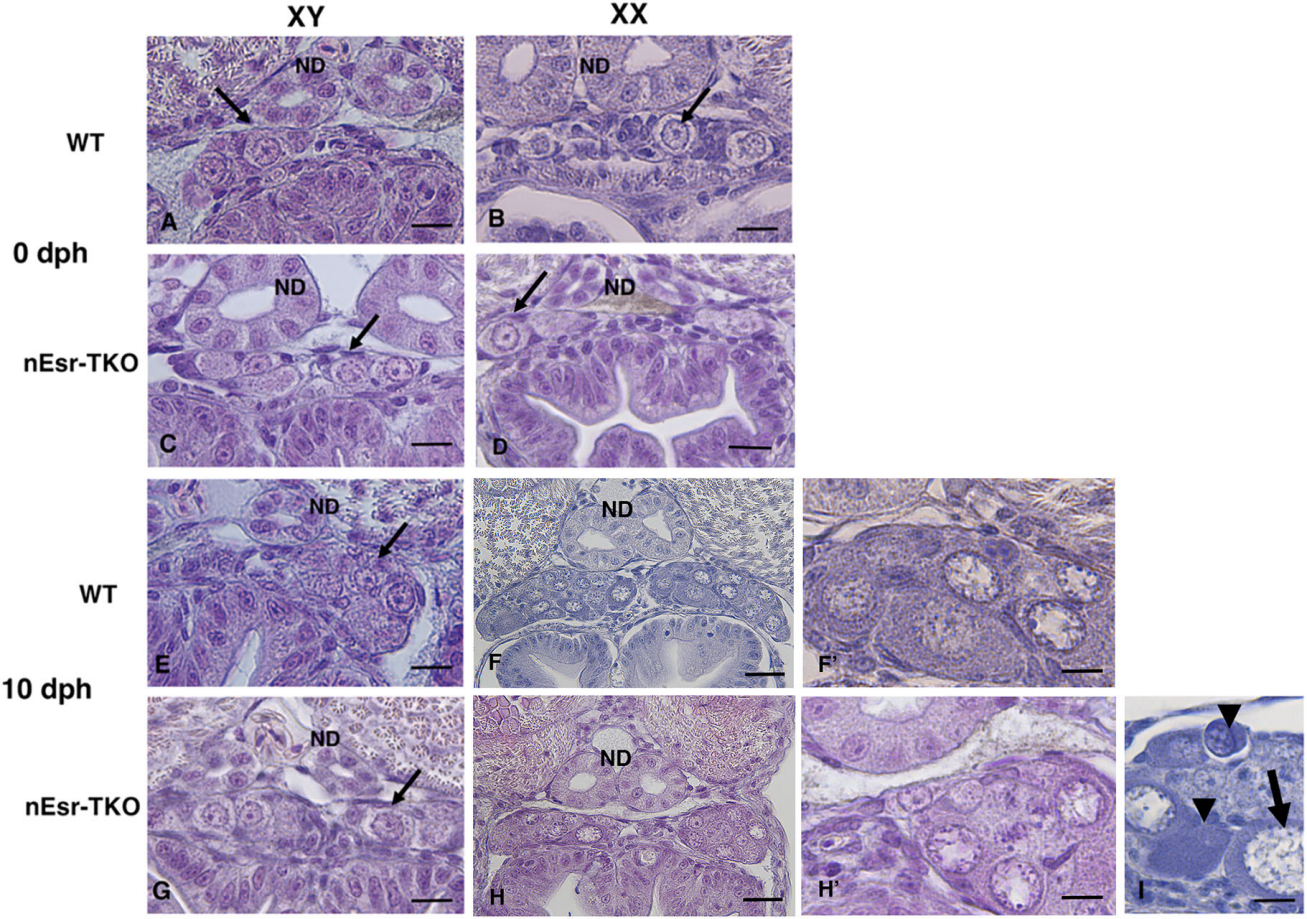
Effects of the triple-knockout of nuclear estrogen receptors (ΔnEsrs) on early gonadal sex differentiation. Herein, gonadal histology in ΔnEsrs at 0 and 10 dph was examined. **(A, B)** Wild-type (WT) XY and XX gonads at 0 dph. **(C, D)** XY and XX ΔnEsrs gonads at 0 dph. **(E)** XY and **(F, F’)** XX WT gonads at 10 dph. **(G)** XY and **(H, H’)** XX Δ*nEsrs* gonads at 10 dph. **(I)** XX ΔnEsrs gonads at 10 dph. ND, nephric duct. Small arrows, gonial germ cell. Large arrows, diplotene oocyte. Arrowhead, apoptotic germ cell. Scale bar, 20 μm.

**Figure 2:**
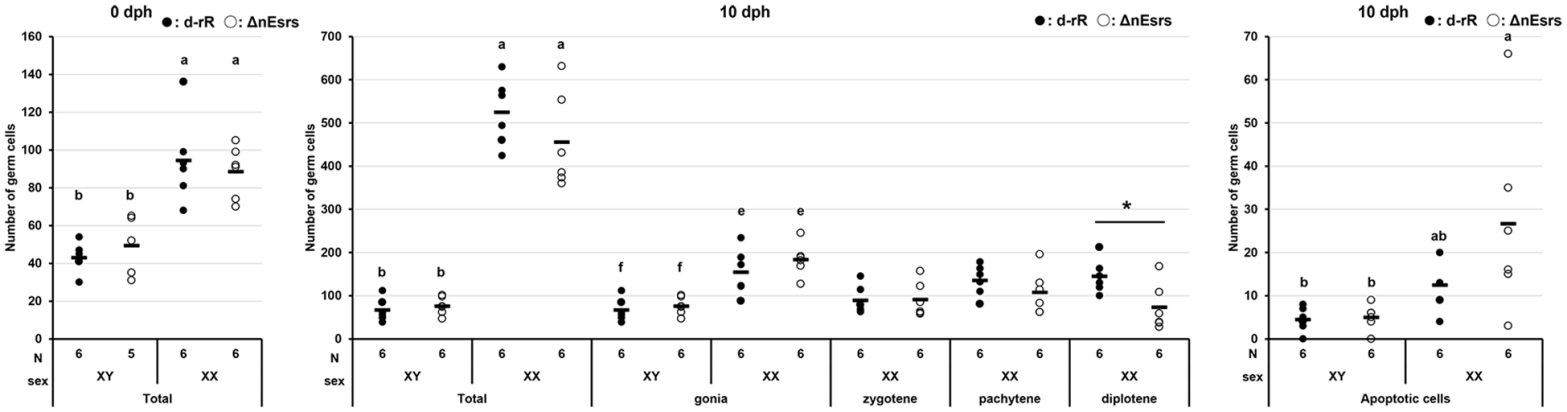
Effects of the triple-knockout of nuclear estrogen receptors (ΔnEsrs) on the kinetics of gametogenesis during early gonadal sex differentiation. Effects of ΔnEsrs on germ cell number for each stage in gametogenesis at 0 and 10 days post-hatching. Each dot represents the germ cell number of each individual. Significant differences between WT and ΔnEsrs were determined by Student’s t-test. *P<0.05. a-f, ANOVA followed by the Tukey–Kramer test was performed for each developmental stage (0 and 10 dph). The significance level was set at 0.05. Total, total number of germ cells. gonia, gonial germ cell. zygotene, zygotene stage of oocytes. pachytene, pachytene stage of oocytes. diplotene, diplotene stage of oocytes. The germ cell showing nuclear condensation was classified as an apoptotic cell. N and Sex indicate the sample number examined, and genetic sex, respectively.

In medaka, sex differentiation is initiated by the sex-determining gene *dmy*, which results in a difference in germ cell numbers between the sexes before hatching, with XX individuals having significantly more germ cells than XY individuals (9). In this study, we investigated the kinetics of germ cell differentiation during early gonadal differentiation in ΔnEsrs. The germ cell differentiation kinetics and gonadal differentiation in XX and XY ΔnEsrs were comparable to those in WT medaka at 0 dph, suggesting that complete deficiency of nEsr does not affect the sex difference in germ cell number and gonadal differentiation at hatching (**Figures 1A–D**, 2: 0 dph). At 10 dph, the total germ cell number in ΔnEsrs mutants was comparable to WT overall, although it showed a decreasing tendency specifically in XX individuals. A significant decrease in the number of diplotene oocytes was observed at 10 dph in XX ΔnEsrs compared to WT, whereas germ cell differentiation kinetics in XY ΔnEsrs were similar to those in XY WT individuals (**Figure 2**: 10 dph). Additionally, apoptotic cells within a single cyst were observed in both XX WT and ΔnEsrs gonads at 10 dph. Based on cell size and their occurrence during the reduction of oocyte numbers, these apoptotic cells were most likely oocytes at pachytene–diplotene stages (**Figures 1H, H’, I**, 2: 10 dph). The number of apoptotic cells in the XX ΔnEsrs was significantly larger than in XY WT and ΔnEsrs, and tended to increase compared to those in the XX WT (**Figure 2**). Together, these findings indicate that nEsr signaling is dispensable for the initial sexual dimorphism as the sex difference in germ cell number, and partially controls oocyte differentiation, such as from pachytene to diplotene.

### Expression of estrogen- and androgen-synthesis related genes and sex differentiation-related genes in ΔnEsrs

XX ΔnEsrs fry caused changes in oogenesis kinetics at 10 dph, such as a decrease in the number of diplotene oocytes (**Figures 1**, **2**). Therefore, we next examined the expression of sex differentiation-related genes during the early stages of gonadal sex differentiation. The genes analyzed included testis differentiation-related gene (*gsdf*), oocyte specific-expressed genes (*figa*, *42sp50*, *bmp15*, and *scp3*), ovary differentiation-related genes (*foxl2* and *amhr2*), estrogen synthesis-related genes (*cyp19a1a* and *cyp19a1b*), androgen synthesis-related gene (*cyp11b*), sex steroid synthesis-related genes (*cyp11a*, *cyp17a1*, and *cyp17a2*), HPG axis genes (*gnrh1, gnrhr1, fshb, lhb, fshr*, and *lhr*), HPS (hypothalamus-pituitary-somatotropic) axis genes (*ghrh, gh1, ghra*, and *ghrb*), and HPT (hypothalamus-pituitary-thyroid) axis genes (*trh* and *tshba*).

**Figure 3:**
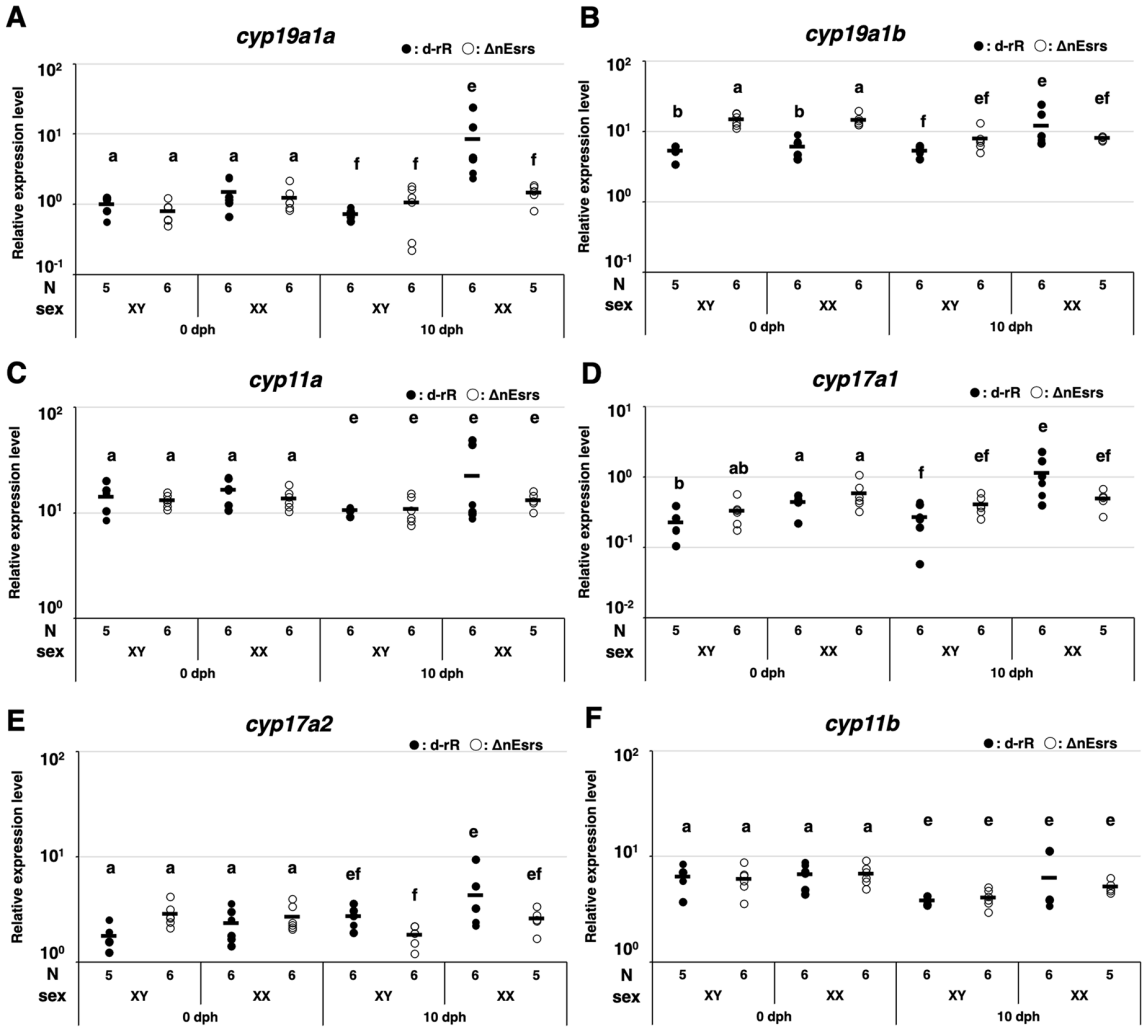
Effects of the triple-knockout of nuclear estrogen receptors (ΔnEsrs) on expression of estrogen and androgen synthesis-related genes at 0 and 10 days post-hatching (dph). The effects of ΔnEsrs on the mRNA expression of estrogen synthesis-related genes [**(A)***cyp19a1a*, **(B)***cyp19a1b*], steroid synthesis-related genes [**(C)***cyp11a*, **(D)***cyp17a1*, **(E)***cyp17a2*], androgen synthesis-related gene [**(F)***cyp11b*] were examined by RT-qPCR. Each plot represents the expression level of an individual, and the horizontal bars indicate the mean values. ANOVA followed by the Tukey–Kramer test was performed for each developmental stage (0 and 10 dph). The significance level was set at 0.05, with significant differences indicated on the plots as a– d d for 0 dph and e– h h for 10 dph. N and Sex indicate the sample number examined, and genetic sex, respectively.

RT-qPCR analysis showed that the expression levels of the estrogen synthesis enzyme *cyp19a1a* were higher in XX WT than in XX ΔnEsrs at 10 dph (**Figure 3A**). *cyp19a1b* mRNA was significantly higher in ΔnEsrs than WT in both sexes at 0 dph, whereas no difference was observed between WT and ΔnEsrs in both sexes at 10 dph (**Figure 3B**). For *cyp11a*, *cyp17a1, cyp17a2, and cyp11b* mRNA, no significant differences were detected between WT and ΔnEsrs (**Figures 3C–F**). In XX at 10 dph, expression of *cyp17a1* and *cyp17a2* tended to be higher in WT than in ΔnEsrs (**Figures 3D, E**).

In HPG, HPT, and HPS axis genes, gene expression examined did not change between WT and ΔnEsrs at 10 dph (**Supplementary Figures S3, S4**). Collectively, this evidence indicates that ΔnEsrs does not affect the expression of genes involved in estrogen and androgen synthesis in XX fry at 10 dph.

**Figure 4:**
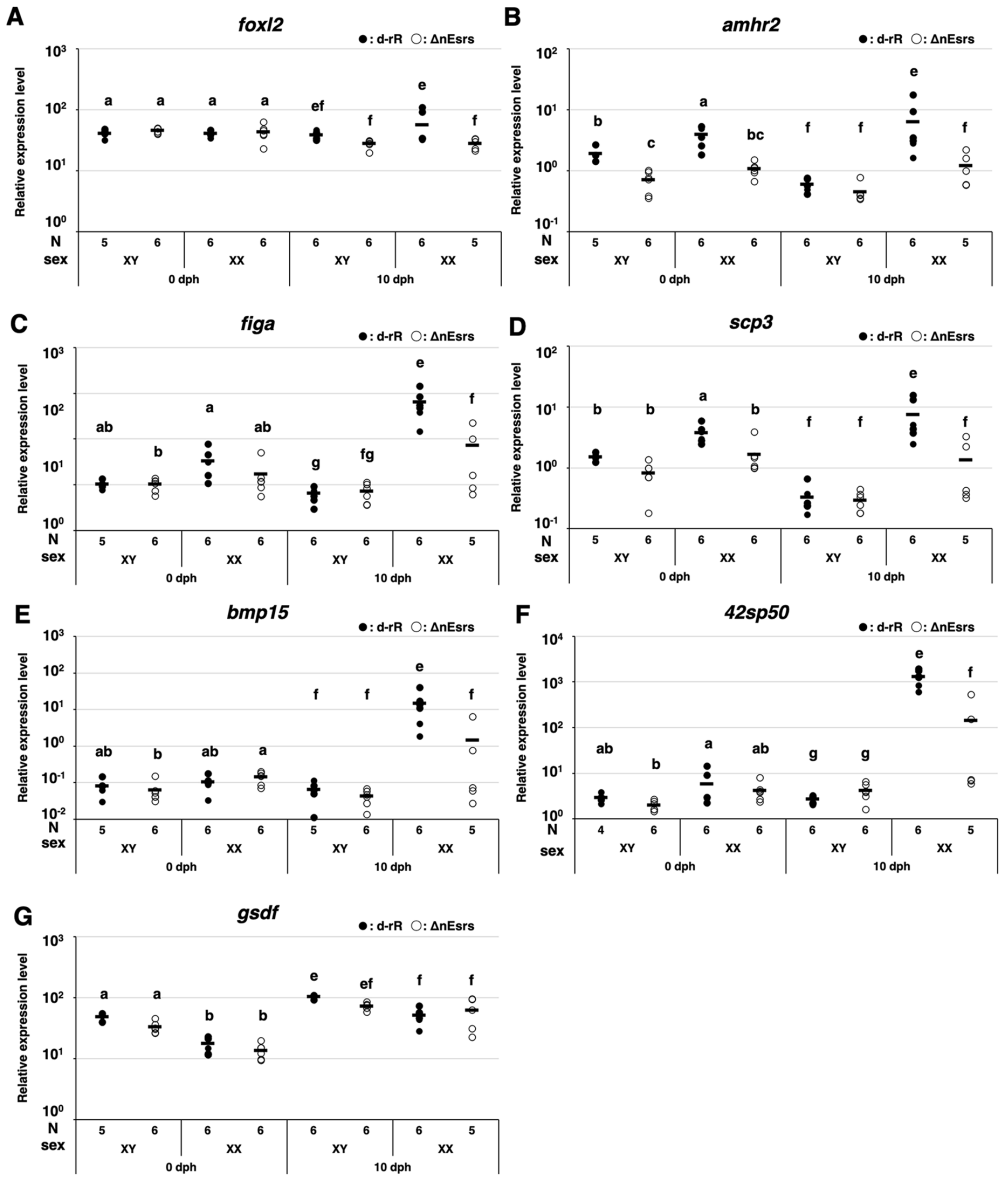
Effects of the triple-knockout of nuclear estrogen receptors (ΔnEsrs) on expression of estrogen and androgen synthesis-related genes and ovary differentiation -related genes, and oocyte-specific expressed genes at 0 and 10 dph. The effects of ΔnEsrs on the mRNA expression of ovary differentiation-related genes [**(A)***foxl2*, **(B)***amhr2*], oocyte specific-expressed genes [**(C)***figa*, **(D)***scp3*, **(E)***bmp15*, **(F)***42sp50*], and testis differentiation-related gene [**(G)***gsdf*] were examined by RT-qPCR. Each plot represents the expression level of an individual, and the horizontal bars indicate the mean values. ANOVA followed by the Tukey–Kramer test was performed for each developmental stage (0 and 10 dph). The significance level was set at 0.05, with significant differences indicated on the plots as a– d d for 0 dph and e– h h for 10 dph. N and Sex indicate the sample number examined, and genetic sex, respectively.

In ovary differentiation-related genes, *foxl2* mRNA significantly decreased in ΔnEsrs compared to WT in XX at 10 dph (**Figure 4A**). The expression of *amhr2* was significantly greater in XX WT than in XY WT at 0 and 10 dph, correlating with the germ cell number in both sexes. In ΔnEsrs, however, no sex difference was detectable at 0 and 10 dph. Additionally, it was significantly lower in XX ΔnEsrs compared to WT at 10 dph, while no difference was observed between WT and ΔnEsrs in XY at 10 dph (**Figure 4B**).

Oocyte-specific expressed genes, *figa*, *scp3*, *bmp15*, and *42sp50* mRNA increased in XX WT at 10 dph compared to those in XX WT fry at 0 dph, corresponding to oogenesis (**Figures 4C–F**). In the XX ΔnEsrs, the expression levels of *figa* at 10 dph, *scp3* at 0 and 10 dph, *bmp15* at 10 dph, and *42sp50* at 10 dph significantly decreased compared with XX WT fry. On the other hand, testis differentiation-related gene, *gsdf*, mRNA was significantly higher in XY than in XX at 0 dph, irrespective of the mutation, and no difference between WT and ΔnEsrs was detected at 0 and 10 dph (**Figure 4G**).

Collectively, nEsr signaling does not affect gonadal sex differentiation up to hatching, including the establishment of germ cell number as the first morphological sex difference. Additionally, nEsr signaling partly promotes oogenic meiosis, the transition from pachytene to diplotene stage, and maintains oogenesis. Nevertheless, complete deficiency of nEsr signaling does not cause feedback regulation for HPG, HPT, and HPS axis gene expression, while it suggests that nEsr signaling upregulates the expression of ovary differentiation related genes, *foxl2* and *amhr2*, and estrogen synthesizing enzyme, *cyp19a1a*, and oocyte expressed genes, *figa*, *scp3*, *bmp15*, and 4*2sp50*.

## Discussion

In the present study, the complete loss of nEsrs did not affect germ-cell number or sex differences at hatching, indicating that maternal or zygotic estrogen signaling via nuclear receptors is dispensable for establishing initial germ-cell dimorphism. In contrast, deficiency of nEsrs markedly impaired the progression from pachytene to diplotene oocytes, demonstrating that estrogen signaling is essential for subsequent meiotic progression. This conclusion was supported strongly by our recent finding that Δcyp19a1s caused a significant decrease in the number of diplotene oocytes (13).

In medaka, an XX/XY system determines sex (22). The sex-determining gene *dmy* is expressed in somatic cells surrounding primordial germ cells at stage (St.) 36. Subsequently, the first morphological sex difference appears as higher germ cell count in XX fry than in XY fry before hatching, St. 38 (7, 9). *Gsdf*, a downstream gene of *dmy*, is dominantly expressed in XY gonads, and its disruption induces excessive proliferation of gonial germ cells, indicating that gsdf suppresses germ-cell proliferation around hatching in XY fry (23, 24). In contrast, the mechanisms of ovarian differentiation including germ cell proliferation and progression in XX individuals remain obscure. Many previous studies have reported the effects of exposure to estrogenic compounds on germ cell number in teleosts including medaka (25). To clarify endogenous estrogenic roles for sex differentiation including maternal (yolk-derived) and zygotically synthesized estrogen, effects of exogenous estrogen have been previously examined; however, the estrogenic role has remained unclear. In some reports, estrogenic compounds induced germ cell proliferation and feminization in XY fish, whereas others suggested that estrogenic compounds did not affect or inhibit it (26–28).

In medaka, fertilized eggs contained E2, and the E2 decreased after 2 days post fertilization (dpf), while E2 is detectable up to 10 dpf (corresponding to 2–3 dph) (29). Together with the expression of nEsrs during embryogenesis in medaka (19), this suggests the possibility that maternal estrogen and its nEsr signaling function in gonadal differentiation and development up to 10 dpf in medaka.

On the other hand, we recently demonstrated that complete loss of aromatase (Δcyp19a1s) did not affect germ cell kinetics or number at hatching, indicating that zygotically synthesized estrogen is dispensable for establishing the initial morphological sex difference (13). Together with our present results of ΔnEsrs, we demonstrate for the first time that endogenous estrogen (maternal estrogen and zygotically synthesized estrogen) via nEsr signaling is dispensable for germ cell kinetics as the first morphological sex difference in medaka, while the possibility of maternal estrogen via membrane Esr, such as *gper1* (29) signaling is not completely ruled out. Importantly, our present results of ΔnEsrs demonstrate that nEsr signaling affects the subsequent progression of oogenesis, particularly from the pachytene to the diplotene stage after hatching. In the present study, loss of nEsrs caused a decrease in diplotene oocytes and tended to increase the number of apoptotic germ cells, probably pachytene oocytes (**Figure 1**, **2**). Similar phenotypes were also observed in Δcyp19a1s medaka, while apoptotic germ cells did not tend to increase (13). Additionally, ΔnEsrs caused upregulation of *cyp19a1a* mRNA at 10 dph, whereas HPG axis gene expression was not controlled by feedback regulation depending on loss of nEsr signaling (**Figures 3**, **4**, **Supplementary Figures S3, S4**). Therefore, the difference in apoptosis between ΔnEsrs and Δcyp19a1s suggests the possibility that maternal estrogen signaling via nEsrs contributes to oocyte survival.

Collectively, these findings demonstrate that nEsr signaling is essential for both the pachytene–diplotene transition and the maintenance of oocytes through local upregulation of *cyp19a1a* mRNA, which likely represents a temporary regulatory mechanism during this pre-pubertal period. Nevertheless, additional estrogenic pathways—such as maternal estrogen or membrane-bound receptors (e.g., *gper1*)—may partially support oocyte survival (30).

Previous reports indicated that loss of *figa* stopped the oocyte differentiation up to pachytene, suggesting that *figα* plays a role in the progression from pachytene to diplotene in medaka and zebrafish, in addition to mice (31–34). In mice, *Figa* knockouts caused all oocytes to undergo apoptosis after birth; this was not observed in teleosts. To date, it has not been clarified whether *figa* expression is controlled by nEsr signaling in teleosts; however, no estrogen-responsive elements (EREs) were detected within the 3 kb upstream region in medaka (data not shown). Therefore, downregulation of *figa* in XX ΔnEsrs may be due to a reduction in diplotene oocytes caused by nEsr deficiency, while it cannot be ruled out that it is regulated via another pathway mediated by nEsr.

Together, our findings propose a refined model of estrogen action in teleost oogenesis: maternal and zygotically synthesized estrogen via nEsr is dispensable for early sex differentiation, whereas zygotic estrogen signaling via nEsr is indispensable for meiotic progression and oocyte survival, revealing an evolutionarily conserved temporal hierarchy of estrogen function across vertebrates. Finally, this study reported the function of estrogen signaling in early gonadal differentiation. Analysis of the ΔnEsrs phenotype in adult fish is currently underway.

## Data Availability

The datasets presented in this study can be found in online repositories. The names of the repository/repositories and accession number(s) can be found in the article/**Supplementary Material**.

## Glossary

*bmp15*: Bone morphogenetic protein 15
*cyp11a*: Cytochrome P450 Family 11 Subfamily A Member
*cyp17a1*: Cytochrome P450 Family 17 Subfamily A Member 1
*cyp17a2*: Cytochrome P450 Family 17 Subfamily A Member 2
*cyp11b*: Cytochrome P450 Family 11 Subfamily B Member
*cyp19a1*: Cytochrome P450 family 19 subfamily A member 1
dpf: days post-fertilization
dph: days post-hatching
*dmrt1*: Doublesex and mab-3 related transcription factor 1
*dmy*: DM-domain gene on the Y chromosome
*ef1a*: Elongation factor 1 alpha
*figa*: Factor in the germline alpha
*fshb*: Follicle stimulating hormone subunit beta
*fshr*: Follicle stimulating hormone receptor
*gh1*: Growth hormone 1
*ghr*: Growth hormone receptor
*ghrh*: Growth hormone releasing hormone
*gnrh*: Gonadotropin releasing hormone
*gnrhr*: Gonadotropin releasing hormone receptor
*gsdf*: Gonadal soma-derived growth factor
HPG axis: Hypothalamus-pituitary-gonadal axis
HPT axis: Hypothalamus-pituitary-thyroid axis
HPS axis: Hypothalamus-pituitary-somatotropic axis
*lhb*: Luteinizing hormone subunit beta
*lhr*: Luteinizing hormone receptor
nEsr: nuclear estrogen receptor
RT-qPCR: reverse transcription-quantitative polymerase chain reaction
*scp3*: Synaptonemal complex protein 3
*trh*: Thyrotropin releasing hormone
*tshb*: Thyroid stimulating hormone subunit beta
