# Supplementary material for: Estrogenic control of germ cell differentiation in medaka: independence of early sex dimorphism from zygotic estrogen and receptor signaling
Source: Front Endocrinol (Lausanne). 2026 Jan 19;16:1769798. doi: 10.3389/fendo.2025.1769798 (PMC12862824; doi:10.3389/fendo.2025.1769798)
Supplement: Supplementary file 1 [file DataSheet1.pdf]

Supplementary Information

Estrogenic control of germ cell differentiation in medaka: independence of early sex dimorphism from zygotic estrogen and receptor signaling

Yuta Sakai-Yamada, [1,2] Taijun Myosho,[1,2] Daichi Kayo,[3] Shinji Kanda,[4] and Tohru Kobayashi[1, 2, *]

[1] Laboratory of Molecular Reproductive Biology, Institute for Environmental Sciences, University of Shizuoka, Shizuoka, 422-8526, Japan

[2] Graduate School of Pharmaceutical and Nutritional Sciences, University of Shizuoka, Shizuoka, 422-8526, Japan

[3] Division of Applied Biosciences, Graduate School of Agriculture, Kyoto University, Kyoto, 606-8502, Japan

[4] Laboratory of Physiology, Atmosphere and Ocean Research Institute, The University of Tokyo, 5-1-5 Kashiwanoha, Kashiwa, Chiba, 277-8564, Japan.

* **Corresponding author**: Kobayashi Tohru, Ph.D.

Laboratory of Molecular Reproductive Biology, Institute for Environmental Sciences, University of Shizuoka, Shizuoka, 422-8526, Japan.

E-mail. tohruk@u-shizuoka-ken.ac.jp

Tel. +81-54-264-5782

Table 1 Primer list for RT-qPCR

Name	Ensembl gene ID	Forward (5'-3')	Reverse (5'-3')	Function
ef1a	ENSORLG00000007614	AGTACGCCTGGGTGTTGGAC	AAACGGGCCTGGCTGTAAG	Internal control
gnrh1	ENSORLG00000014247	CTGGAGGGAAGCGAGAACTG	GACTCCTCCAGGTGGCTCAAG	HPG axis: Gonadotropin releasing hormone signaling
gnrhr1	ENSORLG00000019213	GGTGGCGTGGACCATGAG	GCACTGGGTGAAGTTTGCTG	
fshb	ENSORLG00000001305	CTGCTGTGCCACTTTCAAGG	GGCTAAAACACCAGCCAGGAC	HPG axis: FSH signaling
fshr	ENSORLG00000010178	TGACGTACCCCTCACACTGC	GATGTATTTCGCCATGGGATG	
lhb	ENSORLG00000003553	GAGGGCTGCTCTGGCTGTC	TCCCGGTACGTACACACATTC	HPG axis: LH signaling
lhr	ENSORLG00000010892	TCACCCATCTGCCCTTGAAAC	CCGTGATGCGGTCACTCTG	
cyp11a	ENSORLG00000007190	TTCATCGGATCATGGTGCAG	GCAGCATCTTCAGGTTTGATG	Steroid hormone synthesis: Cholesterol side chain cleavage
cyp17a1	ENSORLG00000019226	GCCTGCTGAAGCAGTGTGTG	TCCCTCTGAACGTGGTCACTG	Steroid hormone synthesis: 17α-hydroxylase/c17-20 lyase
cyp17a2	ENSORLG00000002242	TCCTTCACTCTGTTTGGGGAAG	TCAGCTCGGAGCACAAACTG	Steroid hormone synthesis: 17α-hydroxylase
cyp19a1a	ENSORLG00000002949	GCTGCAGGAGATAGACACCATTG	GCAGGCATTCGTTGATGAAG	Estrogen synthesis (Ovarian type)
cyp19a1b	ENSORLG00000005548	CGTGGAGACAAAGTCACCTTTCAC	CTGTTCCAATCCCCATCCAG	Estrogen synthesis (Brain type)
cyp11b	ENSORLG00000010480	TCAAGAACGGAGAAGAATGGAG	CTTTCGCCACGTCGTCTAGG	Androgen synthesis
bmp15	ENSORLG00000008622	TGCACTACCGACCCACTTCAG	TGCGCAGGTGGATAAAGGAC	Oocyte specific expression (scp3 is meiotic cell- specific expressed gene)
42sp50	ENSORLG00000019066	TTGATCCCAGAAAGCTGGAG	CTCGCTCCCTCTCAGCTTTG	
scp3	ENSORLG00000009617	GAAGGAGCGGATGAATGTGC	TGCTGACTTCCTGTCATGTGG	
figa	ENSORLG00000015451	AATGGTCAACGCCAAGGAAAG	TTGGCTTTTTGTCTGGTTGC	
ghrh	ENSORLG00000022015	GTCAGGTTCCCCGCTCTACC	GGCTCAGCTGGGTCTCTCAC	HPS axis: Growth hormone releasing hormone
gh1	ENSORLG00000019556	GCCCATTTCAGAGCAGATCC	ACAGACAGCTGACAGCAGAAGG	HPS axis: Growth hormone signaling
ghra	ENSORLG00000004053	CCTACTGCGTGCAGCTTCG	AGAGACACTGGTGGGTCAGG	
ghrb	ENSORLG00000014800	AGATGCACGGCATGACCAG	AGCCATCACTGGGAATCTCG	
trh	ENSORLG00000010934	TCATCCTGAGGAGAGCAGAAAG	TGCCATCCAATCCTGTTGTG	HPT axis: Thyrotropin releasing hormone
tshba	ENSORLG00000029251	GGAGAAGCCAGAATGCGACTAC	CAAAATGTCCCTCATGTTGC	HPT axis: Thyroid stimulating hormone

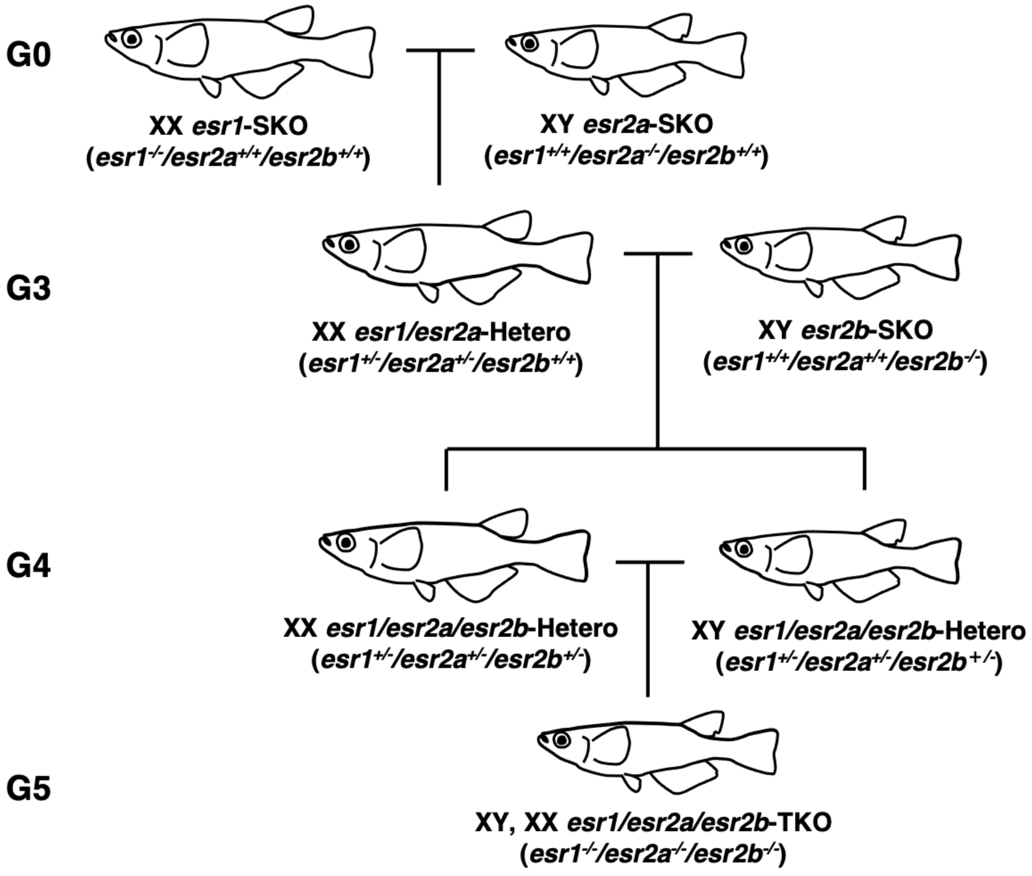

Fig. S1 Establishment of nuclear estrogen receptors (nEsr) double knockouts (DKOs) and a triple knockout (TKO) in the d-rR medaka

nEsr DKOs and a TKO were established using single knockouts (SKOs) of three nEsr in the d-rR medaka.

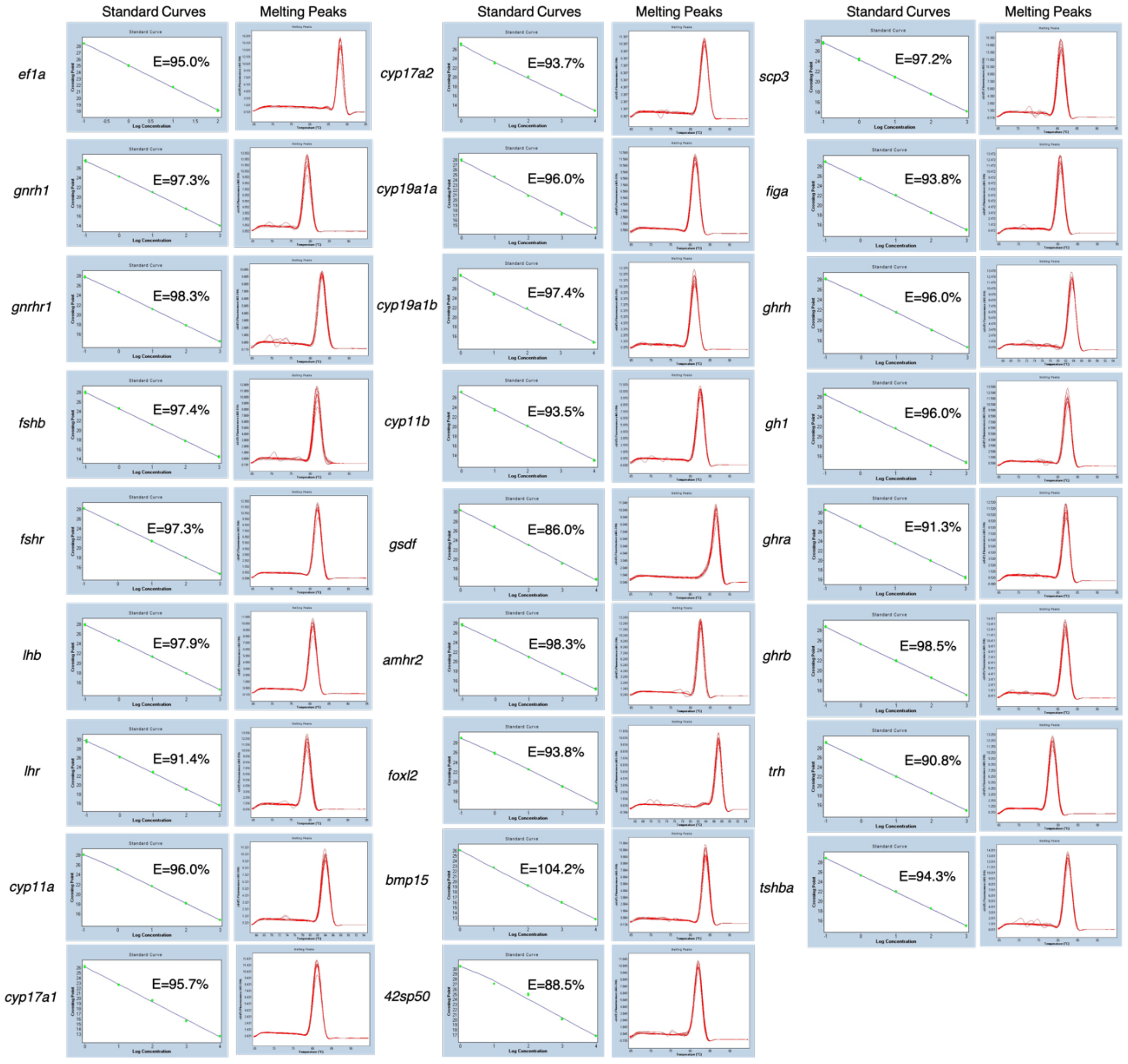

Fig. S2 Specificity of RT-qPCR

Standard and melting curves, and E-values were shown for each mRNA.

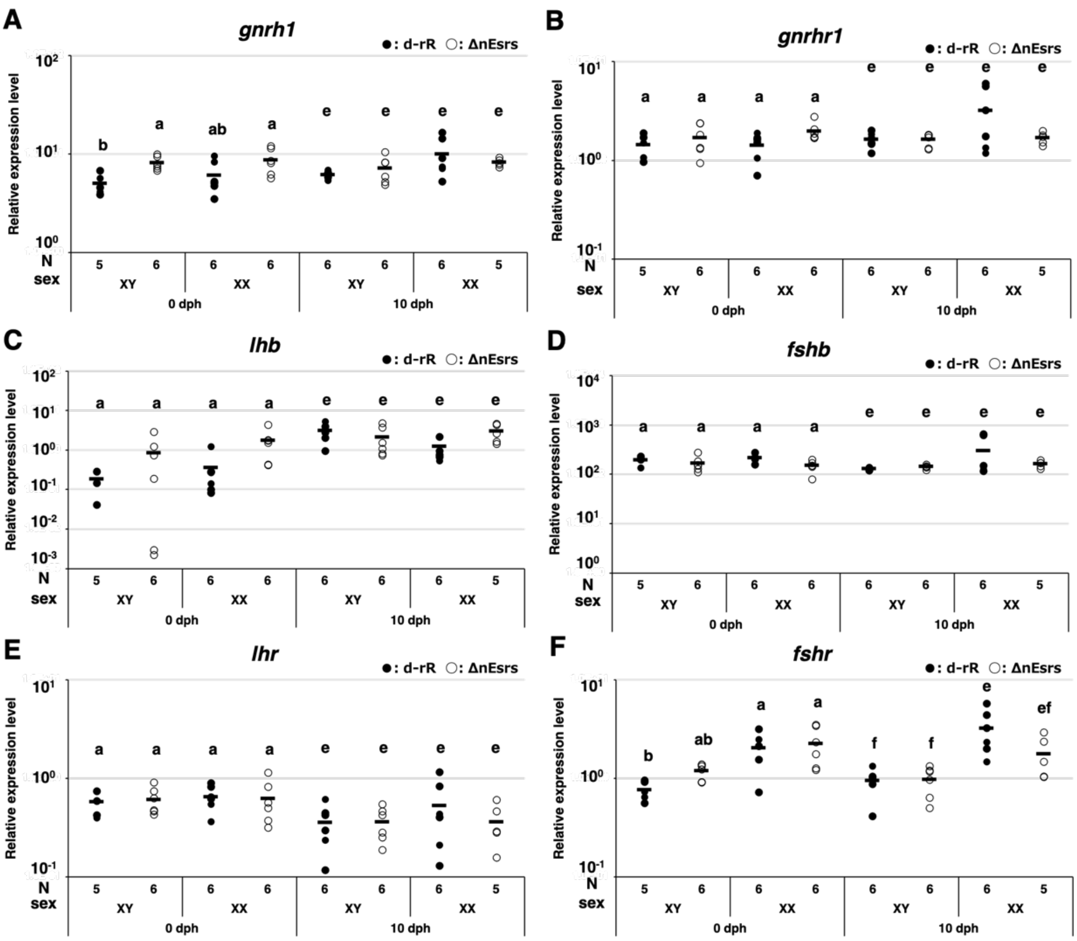

Fig. S3 Effects of triple knockout of nuclear estrogen receptors (ΔnEsrs) on mRNA of Hypothalamus-pituitary-gonadal axis genes

A. *gnrh1*, B. *gnrhr1*, C. *lhb*, D. *fshb*, E. *lhr*, F. *fshr*. Each plot represents the expression level of an individual, and the horizontal bars indicate the mean values. ANOVA followed by the Tukey–Kramer test was performed for each developmental stage (0 and 10 dph). The significance level was set at 0.05.

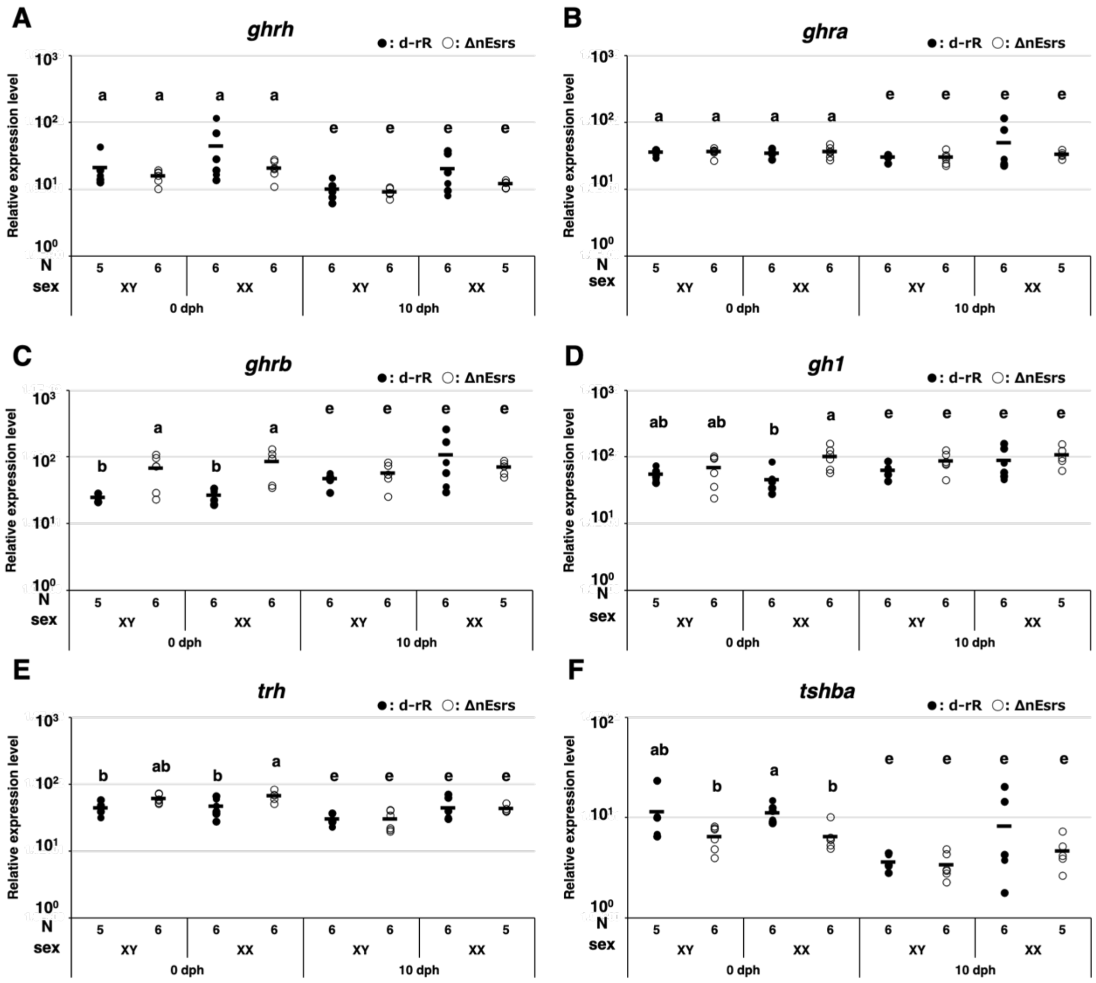

Fig. S4 Effects of triple knockout of nuclear estrogen receptors (Δ*nEsrs*) on mRNA of Hypothalamus-pituitary-somatotropic axis (A-D) and Hypothalamus-pituitary-thyroid axis (E, F) axis genes

A. *ghrh*, B. *ghra*, C. *ghrb*, D. *gh1*, E. *trh*, F. *tshba*. Each plot represents the expression level of an individual, and the horizontal bars indicate the mean values. ANOVA followed by the Tukey–Kramer test was performed for each developmental stage (0 and 10 dph). The significance level was set at 0.05.
